# Decision-making capacity evaluation in older cancer patients: A call to action

**DOI:** 10.1017/S1478951525100564

**Published:** 2025-11-14

**Authors:** Patricia A. Parker, Faith C. Fasakin, Daniel McFarland, Yesne Alici, Liz Blackler, Julia D. Kulikowski, Konstantina Matsoukas, Beatriz J. Korc-Grodzicki

**Affiliations:** 1 Department of Psychiatry and Behavioral Sciences, Memorial Sloan Kettering Cancer Center, New York, NY, USA; 2 Department of Psychiatry, Weill Cornell Medical College, New York, NY, USA; 3 Departments of Medicine, Hematology/Oncology and Psychiatry, University of Rochester Medicine, Rochester, NY, USA; 4 Ethics Committee and Consultation Service, Memorial Sloan Kettering Cancer Center, New York, NY, USA; 5 DigITs, Memorial Sloan Kettering Cancer Center, New York, NY, USA; 6 Geriatrics Service, Department of Medicine, Memorial Sloan Kettering Cancer Center, New York, NY, USA

**Keywords:** Older adults, cancer, decision-making capacity, communication, Shared decision-making

## Abstract

**Objectives:**

Patients’ involvement in the decision-making process is essential for shared decision-making and optimal patient-centered care. However, when there are concerns about a patient’s cognition and judgmen, the complexity of providing patient-centered care increases. It is often necessary to evaluate patients’ decision-making capacity (DMC) to determine whether they are able to make a particular decision or whether to rely on their previously expressed wishes or the patient’s caregivers.

**Methods:**

In this article, we present a case of an older adult with colon cancer who presented to the emergency room.

**Results:**

We describe how multidisciplinary care can enhance the evaluation of DMC and improve quality of care for older patients with advanced cancer.

**Significance of results:**

Multidisciplinary discussions and good communication skills are essential for navigating these complex situations, reducing potential harm and maximizimizing quality of life.

Shared decision-making (SDM) in older adults with cancer is a complex endeavor. In addition to the patient’s wishes and beliefs, numerous factors including cognitive decline, frailty, comorbidities, polypharmacy, and sensory impairment greatly influence treatment decision-making. This is particularly challenging when older adults have difficulties making decisions about their medical care. SDM, a process in which the clinician works in collaboration with the patient to make decisions about clinical care that is consistent with the patient’s preferences, goals, and values, is a key element of patient-centered care (Scholl et al., 2014; Shickh et al., 2023; Zhou et al., 2023). However, when there is concern about a patient’s cognition or judgment, it is essential to evaluate decision-making capacity (DMC) and may be necessary to rely on patients’ caregivers or previously expressed wishes if patients are unable to demonstrate this.

DMC is an adult patient’s ability to understand and process information about their diagnosis, prognosis, and treatment options. Persons with DMC are able to weigh the relative benefits, burdens, and risks of therapeutic options; manipulate medical information in a manner to make treatment choices aligned with personal values; and communicate a consistent choice regarding the decision (Appelbaum and Grisso 1988). Importantly, DMC is specific to a particular decision.

Decisional incapacity is often underrecognized in older adults, and the decision to assess capacity varies depending on the clinician and care environment (Gan et al., 2023; Sessums et al., 2011). The capacity assessment process must account for the serious consequences of consenting to or refusing treatment and should involve consultation with relevant clinical experts (McFarland et al., 2020). While efforts are being made to standardize DMC assessment practices, the process can be optimized by incorporating the perspectives of clinicians from multiple disciplines that have relevant expertise (Charles et al., 2021; National Institute for Health and Care Excellence 2018; Seyfried et al., 2013). We describe the case of an older patient with advanced cancer who presented with a recurrent theme: the difficulties clinicians encounter during SDM, especially when the older adult is cognitively impaired. It illustrates how those difficulties affect the quality of care and may prevent the clinician from paying attention to what matters most to the patient.

## Case description

Mr. Jones is an 85-year-old man with hypertension, diabetes mellitus, depression, and unsteady gait. He was diagnosed with rectal cancer involving the anal sphincter. He consulted a colorectal surgeon who recommended an abdominal perineal resection (APR) and chemoradiation. Mr. Jones was told that the APR would result in an irreversible colostomy. His physical examination showed a palpable anal mass. It was otherwise unremarkable.

Mr. Jones lived alone and paid for help with cleaning and meal preparation. He had no children and identified a nephew as his healthcare agent (HCA).

Mr. Jones adamantly refused to consider a colostomy and declined all treatment for his cancer. He remembered his brother struggling with a colostomy for years before he died. Mr. Jones said, “I would rather die than suffer the way my brother did.” He did not attend any follow-up appointments.

Months later, Mr. Jones’s nephew brought him to the emergency department (ED) confused and experiencing abdominal distention, pain, and emesis. Imaging revealed a large bowel obstruction. The ED clinicians determined that he was not able to demonstrate decision-making capacity (DMC) to consent for surgery. They talked with his nephew about the need for an emergent colostomy as a lifesaving procedure versus end-of-life care. The nephew consented to surgery. After a difficult postoperative period, the patient was discharged to a subacute rehabilitation facility.

## Discussion

This case illustrates a complicated scenario where the patient had a clear preference and rationale for not accepting curative-intent treatment. When treatment is presented to such a patient, the clinician must address what could happen if treatment is not pursued and record a summary of the discussion in the chart. It is crucial to check understanding of the disease and the available treatment options while exploring what matters most to the patient. It is also an opportunity to talk about end-of-life goals of care (GOC). If there is a concern about the patient’s cognition, he should be evaluated for DMC since it may be necessary to rely on a caregiver. Determining DMC can be challenging and time-consuming (Seyfried et al., 2013). Oncologists, geriatricians, and/or psychiatrists all may address dementia, mood disorders, and chemotherapy-related cognitive impairment when caring for older adults with cancer, where informed consent for treatment is imperative (Marron et al., 2020). Evaluating patients for DMC often requires cross-disciplinary collaboration to provide timely patient-centered care (Moye and Marson 2007).

Mr. Jones presented to an ED with his nephew, who reported a new onset of confusion. It is a must to screen/make a diagnosis of delirium on admission and focus on the identification and management of the underlying medical etiologies. If Mr. Jones could have been stabilized and had periods of clear thinking, those should have been utilized to address his wishes.

Mr. Jones had multiple comorbidities. Therefore, accurate understanding of his functional age and frailty status is critical. Functional age is more useful than chronological age to define aging and a prognostic factor of how well he would withstand the rigors of treatment. Frailty is a risk factor for poor tolerance to surgical stress with potentially devastating consequences such as postoperative complications, discharge to a skilled nursing facility, and poor quality of life (QOL; Fried et al., 2001; Makary et al., 2010). It appeared that the preservation of his QOL was what mattered most to Mr. Jones, and a colostomy was incompatible with acceptable QOL for him. His wishes appeared to be consistent over time, and there were no obvious reasons not to respect them.

Cancer-related psychological distress may result in functional decline (Hurria et al., 2009). In addition, the lack of social support would make it difficult for him to remain living independently with a colostomy bag to care for. Placement in a rehabilitation facility followed by long-term care would be the most likely postoperative scenario. Is that something Mr. Jones would agree with? Is his nephew considering becoming his caregiver? Is the nephew’s decision based on his own feelings and beliefs? Are there financial incentives at play? Given these many possibilities, involving an interprofessional team would be invaluable.

Consultation with an Ethics Committee should be considered when the patient’s autonomy is compromised, and the HCA makes decisions inconsistent with the patient’s stated wishes. Autonomy is a fundamental principle of bioethics that prioritizes an individual’s right to make informed decisions about their medical care. A person with capacity has an inherent right to self-determination and can decide whether to accept or decline interventions, even life-saving ones. In this case, it was unclear whether Mr. Jones understood the consequences of his decision, even if it meant a shortened lifespan. In hindsight, the surgical team could have used the opportunity of his outpatient consultation to document his GOC by completing state/institution-approved advanced directives (Comer et al., 2020). To override Mr. Jones’ previous decision simply because he now lacks DMC is ethically fraught and should not be done without thorough discussion involving the institution’s Ethics Committee.

Optimal communication is essential at all encounters. There was a discrepancy between Mr. Jones’ expressed wishes, and his nephew’s consent to surgery. Communication strategies and skills could be used to help disentangle this situation. Checking the nephew’s understanding of his uncle’s wishes, asking open questions to explore how involved he had been in his uncle’s care and if he had been present for any conversations in which his uncle expressed his desire to decline treatment could be helpful. Clinicians rarely receive training on how to best communicate with older adults and their caregivers. Communication skills training programs for interprofessional clinicians have been shown to help them communicate more effectively (Parker et al., 2023; Rosa et al., 2022).

## Conclusion

DMC evaluation is a cornerstone of person-centered care (American Geriatrics Society Expert Panel on Person-Centered Care 2016). An oncology practice built on the foundation of SDM requires a firm understanding of DMC and the deliberate practice of incorporating what matters most to the patient into management decisions. GOC discussions should be an essential and routine part of medical visits. Multidisciplinary discussions and good communication skills are essential to navigating these complex situations and ethical challenges.
